# Eustachian Valve Endocarditis Secondary to Candida glabrata Complicated by Frailty and Acute COVID-19 Infection

**DOI:** 10.7759/cureus.86198

**Published:** 2025-06-17

**Authors:** Brittany L Davis, Ronald T Garry

**Affiliations:** 1 Internal Medicine, NCH (Naples Comprehensive Health) Healthcare System, Naples, USA; 2 Geriatrics, NCH (Naples Comprehensive Health) Healthcare System, Naples, USA; 3 Florida Medical Group, Northwestern Medicine McHenry Hospital, Naples, USA; 4 Internal Medicine, University of Central Florida College of Medicine, Orlando, USA

**Keywords:** bacteremia, endocarditis, eustachian valve endocarditis, fungemia, geriatrics, immunosenescence, older adults

## Abstract

Transesophageal echocardiograms have revealed that the eustachian valve persists in a small proportion of the adult population. Only a few cases of eustachian valve endocarditis have previously been reported in older adults. This case report describes an 82-year-old male patient with eustachian valve endocarditis. He had a remote history of infrarenal abdominal aortic aneurysm post endovascular leak repair, recent septicemia from* Streptococcus mitis *from an infected tooth, frailty, diabetes, and immunosenescence. He was found to have developed bacteremia and fungemia.

## Introduction

Eustachian valve endocarditis is a rare phenomenon, and very few cases have been reported [1]. The eustachian valve, which is found at the junction of the inferior vena cava and right atrium [2], facilitates fetal circulation from the inferior vena cava to the left atrium through the foramen ovale [3]. Because it is an embryological remnant of the sinus venosus valve, it is rarely involved in bacterial endocarditis [4]. However, 4% of adults are found to have a persistent eustachian valve on transesophageal echocardiogram [5] and are thus potentially at risk for eustachian valve endocarditis. Risk factors associated with the development of the disorder include intravenous drug use, cardiac pacemakers, cardiac implants, and central venous lines [6]. In this report, we present a rare case of eustachian valve endocarditis secondary to Candida glabrata.

## Case presentation

An 82-year-old man presented to the emergency department with generalized weakness, a new inability to walk, and urinary frequency that began one week prior to admission. He denied cough, nasal congestion, or headache. His past medical history was notable for Parkinson’s disease, remote infrarenal abdominal aortic aneurysm post-repair with iliac endograft, and repair of endoleak 12 weeks prior to this admission. He also had remote coronary artery disease post-stenting, frailty, and dental implants. The patient had *Streptococcus mitis/oralis* bacteremia secondary to a dental infection 18 weeks prior to this admission, and it was treated with 4-6 weeks of ceftriaxone 2 g/d intravenously(IV). Removal of the infected tooth had been recommended, but the patient declined extraction.

Based on laboratory results (Table 1), a urinary tract infection was suspected.

**Table 1:** Urinalysis

	Results	Reference
Leukocyte esterase	+3	Negative
Glucose	+4	Negative
Blood	+2	Negative
Nitrite	Negative	Negative

The patient was started on intravenous ceftriaxone 1 g/d IV for a presumed genitourinary source, and blood and urine samples were collected for culture. A chest X-ray did not reveal any abnormalities. The patient was screened in the emergency room for viral respiratory pathogens, and the test was positive for COVID-19. The infectious disease consultant recommended remdesivir for three days to prevent progression to severe disease [7]. The patient did not need supplemental oxygen. Urine culture results came back negative on day 2, and antibiotic therapy was stopped.

The patient’s hospital course was complicated by progressive clinical deterioration and delirium that was initially attributed to the use of remdesivir, which was subsequently stopped. One of three blood cultures was positive for *Parvimonas micra*. Repeat blood cultures were ordered, and the patient was restarted on intravenous ceftriaxone. *Candida glabrata* grew in two of the two repeated blood cultures on hospital day 5. The patient was started on micafungin 100 mg/d IV.

**Figure 1:**
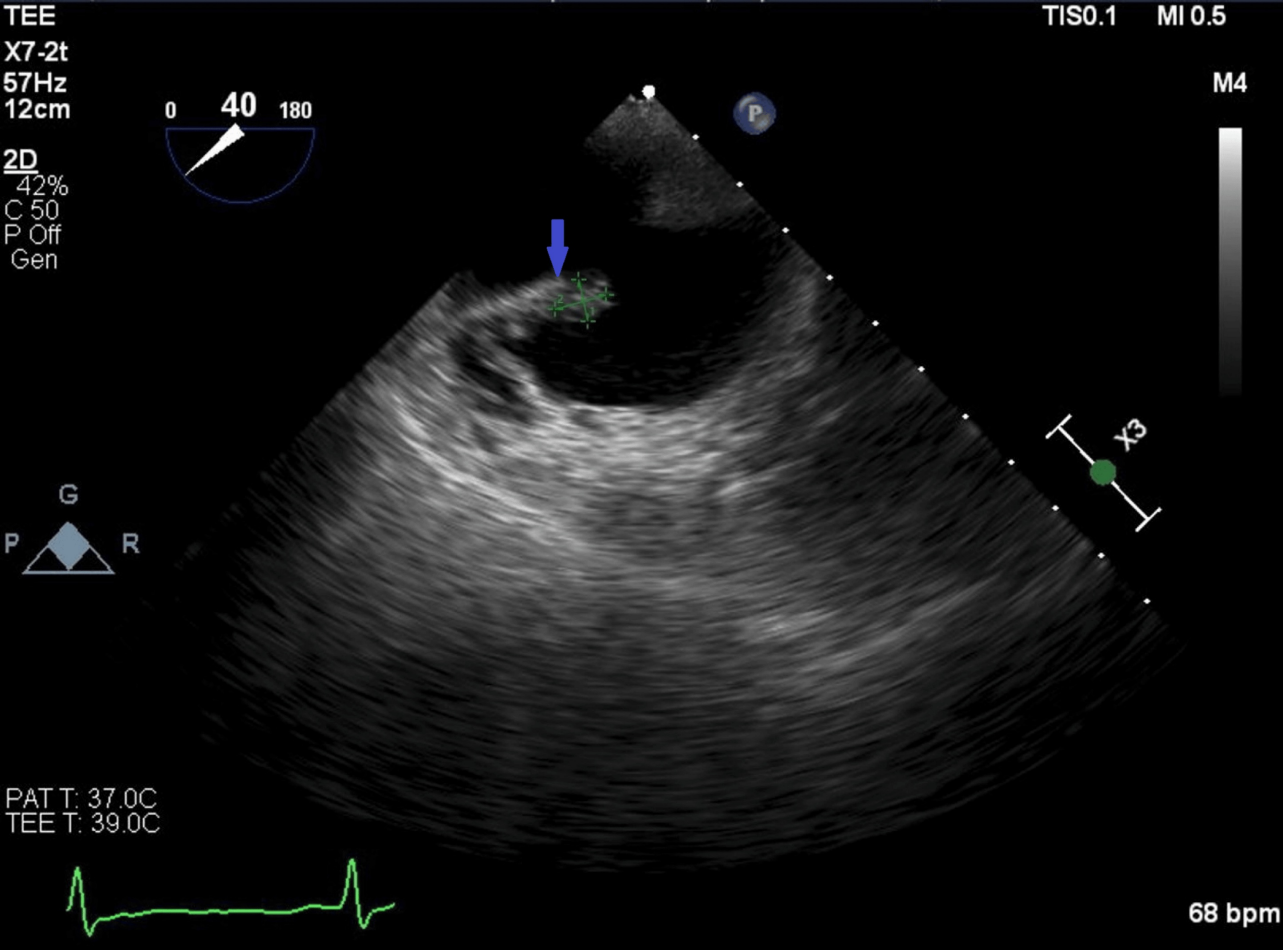
Transesophageal echocardiogram showing the eustachian valve (blue arrow) and mass corresponding to suspected vegetation on the eustachian valve (green highlight).

**Figure 2:**
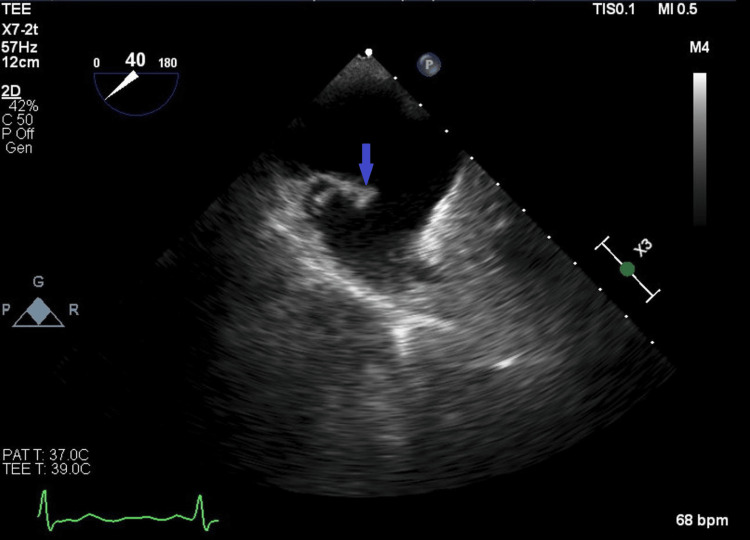
Transesophageal echocardiogram showing the eustachian valve (blue arrow)

A transthoracic echocardiogram was ordered for endocarditis evaluation, and no vegetation was noted. However, the transesophageal echocardiogram revealed a small mobile mass on a prominent eustachian valve that was highly suspicious for eustachian valve endocarditis (Figure 1 and Figure 2). The patient’s Parkinson’s disease and a clinical frailty score of 7 made his prognosis poor [8]. After multidisciplinary coordination, he was discharged to hospice care and later died.

## Discussion

Eustachian valve endocarditis is a rare disease, with risk factors such as intravenous drug use, pacer wires, cardiac implants, central venous lines, congenital heart disease, and endovascular grafts [9]. In this case presentation, the patient had previously had an iliac endograft placed after the repair of an abdominal aortic aneurysm. Common bacteria involved in eustachian valve endocarditis are methicillin-resistant *Staphylococcus aureus* and methicillin-sensitive *Staphylococcus aureus*, which account for 63% of cases [10]. In the current case, blood cultures were positive for *Parvimonas micra *and *Candida glabrata*, and the infections were treated with ceftriaxone and micafungin, respectively. *Candida glabrata* only accounts for 1%-2% of all endocarditis cases and is associated with a mortality rate as high as 80% [11]. This case highlights the importance of using a transesophageal echocardiogram to assess the eustachian valve during persistent or recurrent bacteremia. A transesophageal echocardiogram is extremely sensitive for finding eustachian valve endocarditis [6], which may go undetected on a transthoracic echocardiogram, and it should not be delayed. Treatment with culture-sensitive and specific antimicrobials for four to six weeks is recommended [12] while complications such as heart failure, valvopathy, or cardiac abscess may require more invasive cardiovascular intervention [13].

Other factors highlighted in this case are immunosenescence and frailty. Frailty is increasingly becoming an essential assessment tool in cardiology to predict patient outcomes and select proper interventions [14]. These concepts must be considered when caring for the geriatric population.

## Conclusions

This case highlights the rare phenomenon of eustachian valve endocarditis and its atypical presentation in an older adult. It is important when considering endocarditis as a diagnosis in an older adult to inspect all cardiac valves. Frailty and immunosenescence play a significant role in older adults, helping explain this rare presentation and ultimately guiding decisions toward nonaggressive care, given the poor prognosis and the patient's wishes.
